# Inter-brain network underlying turn-based cooperation and competition: A hyperscanning study using near-infrared spectroscopy

**DOI:** 10.1038/s41598-017-09226-w

**Published:** 2017-08-17

**Authors:** Tao Liu, Godai Saito, Chenhui Lin, Hirofumi Saito

**Affiliations:** 1 School of Management, Zhejiang University, Hangzhou, 310058 China; 2 Department of Cognitive Informatics, Graduate School of Informatics, Nagoya University, Furo-cho, Chikusa-ku, Nagoya, 464-8601 Japan; 3 Department of Psychology, Sun Yat-Sen University, Guangzhou, 510275 China; 4 Department of Psychology, Graduate School of Arts and Letters, Tohoku University, 27-1 Kawauchi, Aoba-ku, Sendai, 980-8576 Japan

## Abstract

The present study examined neural substrates underlying turn-based cooperation and competition in a real two-person situation. We simultaneously measured pairs of participants’ activations in their bilateral frontal, temporal, and parietal regions using a 96-channel near-infrared spectroscopy (NIRS) system, when participants played a turn-taking disk-game on a computer. NIRS data demonstrated significant inter-brain neural synchronization (INS) across participant pairs’ right posterior superior temporal sulcus (pSTS) in both the cooperation and competition conditions, and the competition condition also involved significant INS in the right inferior parietal lobule (IPL). In addition, competitive dyads’ INS in the bilateral inferior frontal gyrus (IFG) may play as a role of mediation in relationship between their empathy score and disk-manipulation latency, but cooperative dyads’ INS did not. These results suggest that first the right pSTS may be commonly involved in both cooperation and competition due to task demands of joint attention and intention understanding, while the right IPL may be more important for competition due to additional requirements of mentalizing resources in competing contexts. Second, participants’ empathy may promote INS in the bilateral IFG across competitors, and in turn affect their competitive performance.

## Introduction

Turn-based interaction is a basic mode of human social behavior, in which people normally taking different roles perform complementary or opposing actions in a turn-taking style^1–3^ (e.g., playing a chess game). The quality of such interactions influences not only individual performance but also our social lives. However, neural substrates of turn-based interaction are still little understood due to the complexity of dynamic interactive contexts and the limitations of brain-imaging techniques^3^. Recently, the hyperscanning technique provides us an opportunity to measure brain activities of two or more persons simultaneously^4^, taking “an important step forward in social neuroscience”^5^, i.e., shifting from an experimental single-brain to a natural multi-brain frame^6, 7^.

Previous hyperscanning studies have investigated neural correlates of concurrent interaction, in which dyadic participants performed tasks requiring body-movement synchrony^8–10^. For example, using an EEG hyperscanning technique, several studies have measured participants’ activities in finger movement/tapping tasks^11, 12^ and music-playing tasks^13^, revealing inter-brain neural synchronization (INS) across interacting members in their right fronto-parietal regions. Similarly, using an fMRI hyperscanning technique, Koike *et al*.^14^ have examined neural substrates of shared attention in a real-time mutual gaze task, and demonstrated INS in the right inferior frontal gyrus (IFG).

Since body-movement synchrony is particularly important for concurrent cooperation, these studies have mainly focused on the INS in cooperative tasks. Concerning concurrent competition, Cui *et al*.^9^, using a near-infrared spectroscopy (NIRS) hyperscanning technique, measured participants’ prefrontal activations in both cooperative and competitive interactions, when they performed a key-press task. Specifically, participant dyads were asked to press two keys at the same time or as fast as possible to defeat their opponent. The NIRS result revealed increased INS values in the right superior frontal cortices during the cooperation but not the competition condition. Taken together, previous hyperscanning studies of concurrent interaction have demonstrated an essential role of the right fronto-parietal regions in cooperative interactions, while there may be little INS in competitive interactions.

In contrast to concurrent interaction, Liu and colleagues^1^ in a NIRS-based hyperscanning study, demonstrated a significantly increased INS value in the right IFG only during competition, when participant pairs taking different roles played a turn-taking disk game. These inconsistent results between the concurrent and turn-based tasks may come from the different task requirements. In both the concurrent cooperation and turn-based competition, participant pairs needed to understand and predict their partner’s body movements or their opponent’s way of thinking about each tactical move, and correspondingly adjust their own actions. That is, the concurrent cooperation and the turn-based competition are inherently *interdependent* tasks and thus shows significant INS across the dyads. However, in the concurrent competition by Cui *et al*.^9^, interacting dyads’ behaviors were *independent* which would not lead to increase of the INS values.

**Figure 1:**
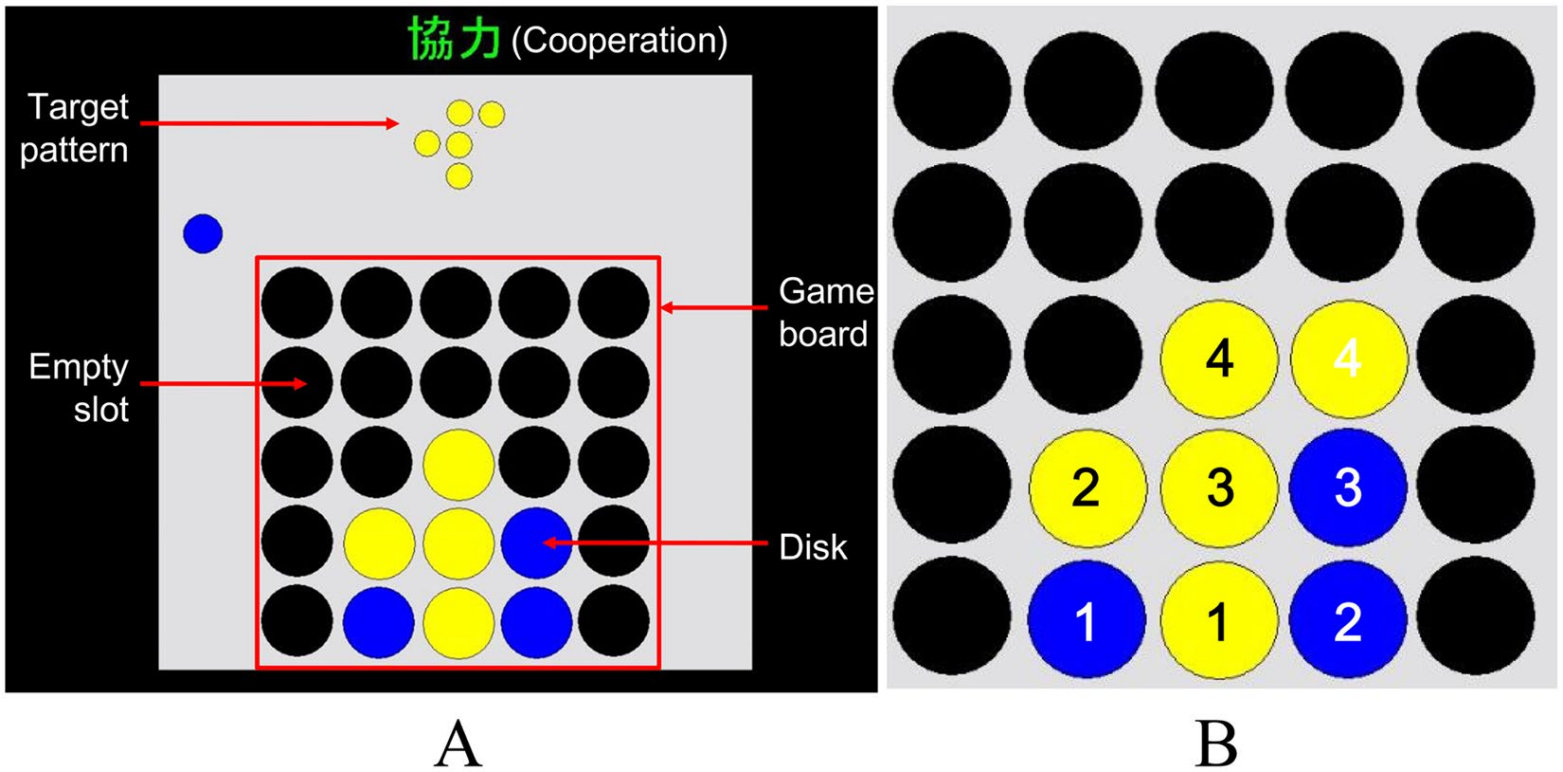
Explanations on (**A**) the turn-taking disk-game task and (**B**) the modification of the game-ending rule in the present study. The left illustration shows a clip from a cooperative game (the arrows and words were not displayed in the experimental game). The small blue disk above the game board initially appeared on the top-left side, indicating that it was the Blue player’s turn to play. Participants could only control the horizontal left and right moves of the disk using a keypad to reach the desired column. The Yellow player always took the initial move, and the Blue player followed. The numbers in the right illustration indicate the orders of the moves of the Yellow and Blue players.

Although Liu *et al*.^1^ did not find INS in the cooperation condition, it is premature to rule out INS in turn-based cooperation. For example, another NIRS hyperscanning study has reported INS in the right prefrontal cortices when participant dyads played a cooperative wooden-block game^15^. Thus not finding any INS in cooperation by Liu *et al*.^1^ may have resulted from the different game design. As Fig. 1A illustrates, in their disk-game participant pairs were randomly assigned to either one of two roles: a Builder controlling yellow disks to copy a target pattern made by five yellow disks, and a Partner controlling blue disks to aid in (cooperation) or to obstruct (competition) the Builder’s task. Each game consisted of eight turns, in which participant dyads played in a turn-taking style (four turns per participant). The Builder always took the initial move, and the Partner followed. A yellow or blue disk appeared automatically every 2 s on the top-left side of the game board, indicating whose turn it was to play. Participants could control only the horizontal left and right moves of the disk using a keypad to reach the desired column within 2 s. The disk then dropped into the lowest available empty slot in the column as if under the force of gravity.

In this game-design the Builder can be considered as the main actor and the Cooperator (i.e., the Partner during cooperation) as the subordinate by default, which in turn may diminish the Cooperator’s motivation and desynchronize their brain activations. To address this issue, the present study modified the original turn-taking disk-game in terms of the experimental instructions and the game-ending rule. First, instead of the Builder-Partner role assignment, participants in the present study were instructed to copy the target pattern collaboratively as a team in a cooperation condition or to occupy as many target positions as possible to defeat the partner in a competition condition. Second, although each participant was still given four disks in one game, in the present cooperative game the color of the fourth blue disk was automatically changed from blue to yellow after the blue-player’s final move, forming five yellow disks, to facilitate copying the target pattern (see Fig. 1B and supplementary video clip). These two modifications assigned equivalent roles to participant pairs and encouraged the blue-players to engage in the game actively, avoiding the demotivation that occurred in the original cooperative game by Liu *et al*.^1^.

Concerning regions of interest (ROIs), previous studies have demonstrated that, for interpersonal interactions^1, 16–18^, understanding others’ emotions, actions, and intentions is a fundamental requirement which is associated with two functional networks, i.e., the theory-of-mind (ToM) network and the mirror neuron system (MNS) network. The theory-of-mind is referred to as the ability of attribution of intention to others^19^. That is, ToM functions to read others’ minds via cognitive processing of inference based on schema stored in our memory. The ToM network mainly consists of medial prefrontal cortex, the temporal pole, and the posterior superior temporal sulcus (pSTS)^20–22^. In contrast, the human mirror neuron system network is defined by the cortical areas which are activated not only during action execution, but also while observing somebody else performing the same or a similar action^23^. Thus MNS, mainly including the IFG and the inferior parietal lobule (IPL), enables an individual to understand others’ actions and intentions via embodied simulation. Taken together, both ToM and MNS are critical for social interaction^24–29^.

**Figure 2:**
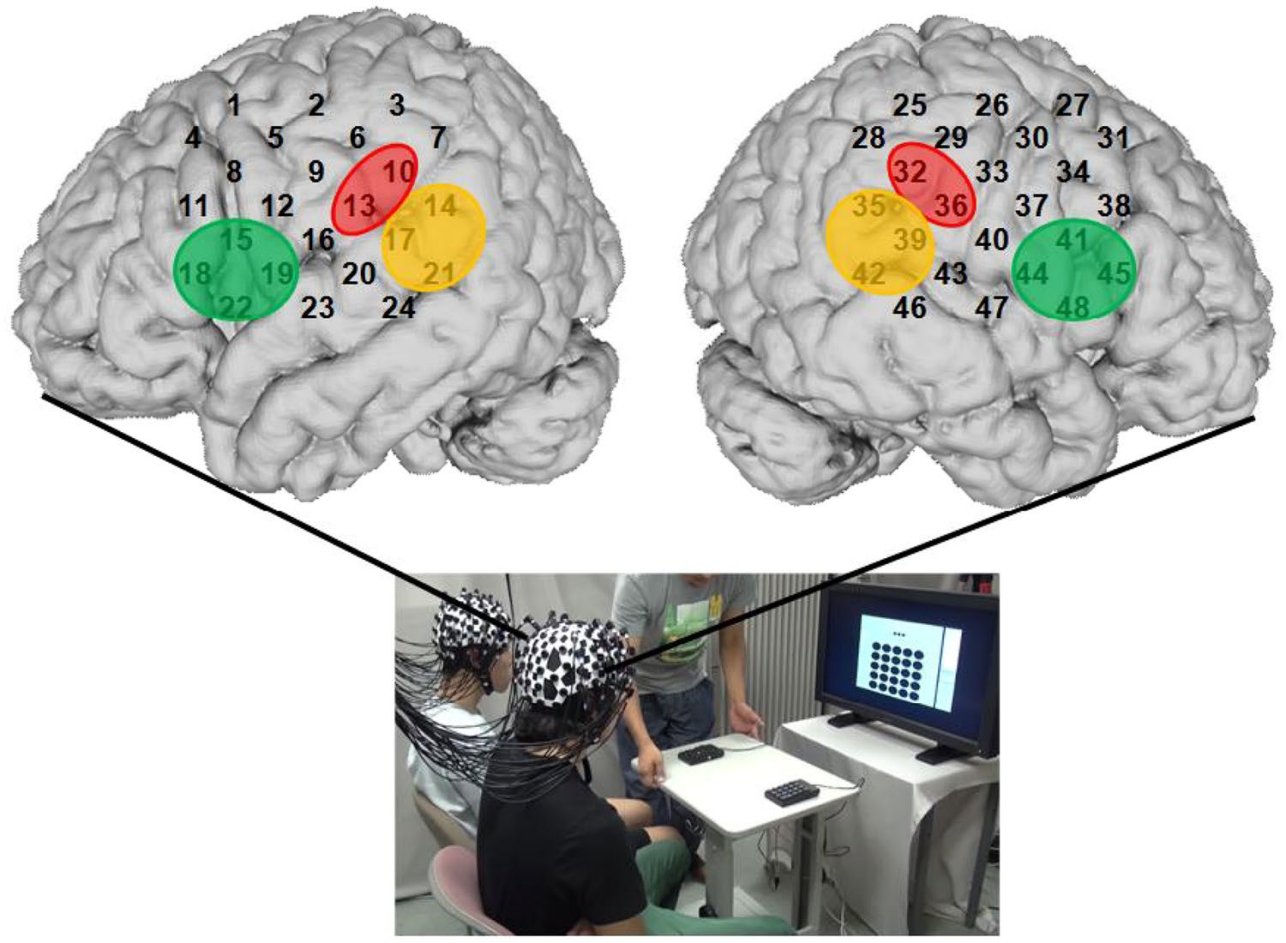
Positions of the 48 channels on one participant’s left and right hemispheres. The bottom picture shows the experimental setting in the present study. The green, red, and yellow overlays cover the bilateral IFG, IPL, and pSTS, respectively. IFG: inferior frontal gyrus; IPL: inferior parietal lobule; pSTS: posterior superior temporal sulcus.

In the present study, we simultaneously measured participant pairs’ activations in the bilateral fronto-tempo-parietal regions (i.e., IFG, IPL and pSTS) as ROIs using a 96-channel NIRS system (LABNIRS; Shimadzu Co., Japan). Figure 2 illustrates positions of the NIRS channels for one participant. Specifically, channels 15, 18, 19, and 22 approximately covered the left IFG, channels 14, 17, and 21 covered the left pSTS, and channels 10 and 13 measured activations in the left IPL. In the right hemisphere, homologous channels measured the right IFG, pSTS, and IPL, respectively (see Methods section for details including the confirmation of the channel locations).

The main hypothesis was two-fold. First, the Yellow-Blue pairs would show a significant INS in the fronto-tempo-parietal regions under both the cooperation and competition conditions, due to *interdependent* features of the present modified disk-game. In addition, since competition involves a clear self-other distinction and requires additional mentalizing resources associated with the right IPL^16^, the INS value in the right IPL would be higher in the competition than in the cooperation condition. Second, since previous studies have reported that empathy is a critical factor modulating human interactive behaviors^13, 30, 31^, we hypothesized that participants’ empathy scores would be positively correlated with both the INS values in the ROIs and their game performance as measured by the error rate and the latency for disk manipulation (see Methods).

## Results and Discussion

### Empathy trait

Participant’s empathy trait was assessed by a four-scale (1: strongly disagree to 4: strongly agree) questionnaire of the Interpersonal Reactivity Index^32^, which was translated into Japanese by Sakurai^33^. The empathy questionnaire consists of 28 items, assessing four aspects of the empathy trait: Perspective-taking (PT), Fantasy (F), Empathic Concern (EC), and Personal Distress (PD). Because the present turn-taking disk-game requires participants to actively understand the partner’s actions and intentions, we mainly focused on the participants’ PT and EC scores. As expected, the *t*-test did not reveal a significant difference between either the Yellow and Blue players’ PT scores [Yellow: 19.14 ± 3.37, Blue: 18.64 ± 4.79; *t*(21) = 0.36, *p* = 0.72] or the EC scores [Yellow: 20.05 ± 3.17; Blue: 19.68 ± 4.50; *t*(21) = 0.27, *p* = 0.79].

### Behavioral performance

To assess participants’ behavioral performance, we calculated the mean error rate (%) and mean latency for disk manipulation (ms) in the cooperation, the competition, and the independent condition (in which one participant played the game under observation by the other participant as a control condition to determine the homogeneity of the brain activity in the two groups of participants), respectively. An error was recorded if Yellow or Blue players disrupted the progress of making the target pattern in the cooperation condition or helped the opponent to achieve the target pattern in the competition condition. The latency was defined as the duration from onset of each turn when the small yellow or blue disk appeared on the top left side of the game board as shown in the Fig. 1A until the participants pressed the key on the keypad to move their disk.

**Figure 3:**
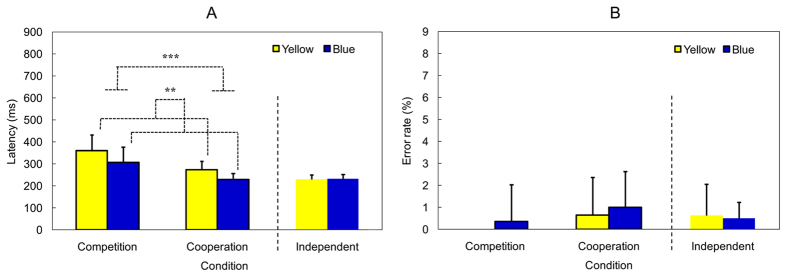
(**A**) Mean manipulation latency and (**B**) mean error rate in the competition, cooperation, and independent conditions. The error bars represent standard deviation. ** and *** indicate *p* < 0.01 and *p* < 0.001, respectively.

Figure 3 illustrates the mean manipulation latency (ms) and the mean error rate (%) in the three conditions. The error rates of both Yellow and Blue players were less than 1%, indicating that participants understood the instructions and played the game effectively. Concerning the manipulation latency, there were significant main effects of Role [*F*(1,42) = 11.47, *p* = 0.002, *η*_*p*_^2^ = 0.21] and Condition [*F*(1,42) = 86.31, *p* = 0.001, *η*_*p*_^2^ = 0.67], and no significant interaction [*F*(1,42) = 0.23, *p* = 0.64]. Simple main effect tests demonstrated that participants used more time for their first move (i.e., the first key press) in each turn during competition (332.59 ± 10.75) than during cooperation (250.10 ± 5.17). Moreover, the latency in Yellow players (first-player: 315.63 ± 10.14) was significantly longer than that in Blue players (second-player: 267.05 ± 10.14) regardless of the cooperation and competition conditions. The latency result may suggest that the cognitive load of the competitive game was higher than that of the cooperative game. That is, the latency difference between the first- and second-players may indicate the so-called “First Move Advantage” in chess games in which the first-player may have more options than the second-player.

### Intra-brain activation

To test homogeneity of the Yellow and Blue players, we first examined their activation differences in the independent condition. As expected, *t*-test analyses revealed no difference in any of the ROIs. A mixed two-way ANOVA [Role (Yellow vs. Blue) × Condition (Competition vs. Cooperation)] was then conducted, and demonstrated significant main effects of Role in the bilateral pSTS [left: Ch14, 17, 21; right: Ch35, 39, 42] and the right IFG [Ch41, 45, 48] (see Table 1 for details). In addition, a significant main effect of Condition was found in the right IPL [Ch36: *F*(1,42) = 9.22, *p* = 0.004, *η*_*p*_^2^ = 0.18], and the right pSTS [Ch39: *F*(1,42) = 5.47, *p* = 0.024, *η*_*p*_^2^ = 0.12]. There were no significant interactions in any of the ROIs.

**Table 1:** Result of significant main effect of Role in the analysis of two-way ANOVA [Role (Yellow vs. Blue) × Condition (Competition vs. Cooperation)].

ROI	pSTS						IFG		
Hemisphere	Left			Right			Right		
Channel	Ch14	Ch17	Ch21	Ch35	Ch39	Ch42	Ch41	Ch45	Ch48
F	4.10	7.34	16.60	5.67	6.61	13.74	9.21	12.81	5.53
*p*	0.050	0.010	0.001	0.022	0.014	0.001	0.004	0.001	0.023
*ηp2*	0.09	0.15	0.28	0.12	0.14	0.25	0.18	0.23	0.12

Note: pSTS, posterior superior temporal sulcus; IFG, inferior frontal gyrus.

**Figure 4:**
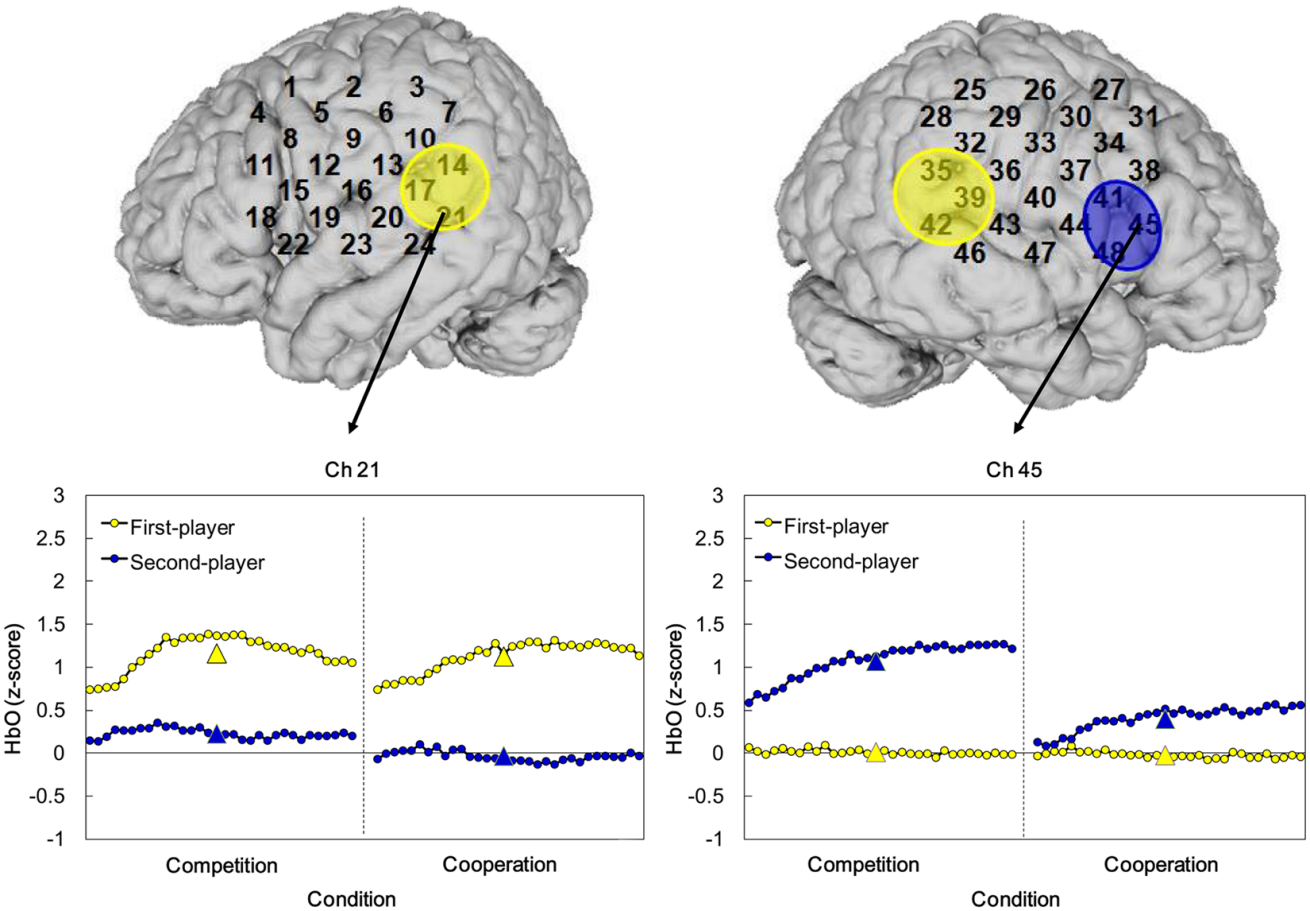
The ROI channels showed statistically significant main effects of role [Yellow (first-player) versus Blue players (second-player)] regardless of the competition and cooperation conditions (*p* < 0.001). The yellow and blue triangles represent the mean Oxy of the Yellow and Blue players, respectively. The yellow overlays cover the bilateral pSTS showing higher activations in the first-players than in the second-players, and the blue overlay covers the right IFG, which conversely showed higher activation in the second-players. Two samples of the activation patterns in selected channels are illustrated at the bottom. HbO: concentration changes of oxygenated hemoglobin.

Simple main effect tests revealed significantly higher right IPL and pSTS activations in the competition than in the cooperation condition, suggesting that greater task demands are involved in competition. The result is consistent with our behavioral data of latency and the evidence from the fMRI study by Decety *et al*.^16^, confirming validity of the present NIRS data. Concerning the role effect, regardless of the interaction types, the first-players (Yellow) showed higher activations in the bilateral pSTS than the second-players (Blue), who conversely yielded higher activation than the first-players in the right IFG (see Fig. 4 for examples). The pSTS is associated with joint attention^34^ and mentalizing^35^, and anatomically adjoins the visual areas. Thus, the first-players may pay more attention to the disk game than the second-players, or consider how to move disks from both the viewpoint of their partner and their own. This result is consistent with the latency data showing a significantly longer time for their action plan as the first-players. Concerning the second-players, since their disk-moves were limited by those of the first-players, they may focus on understanding the first-players’ moves and then adopting their own moves correspondingly, involving higher activation in the right IFG^25, 36, 37^.

### Inter-brain neural synchronization

In the present study, we adopted three steps to assess INS across the Yellow-Blue pairs in the competition and cooperation conditions using General Linear Models. First, we independently analyzed the relationship between two NIRS time series of each pair of participants over multiple consecutive 16 s game rounds, and tested for temporal modulation at the rate of the game rounds. Specifically, a linear regression analysis was conducted with game timing as a regressor, and the ROI channels that showed significant positive relationships between the two NIRS time series of the Yellow-Blue pairs (i.e., the INS indexed by the Beta value) were obtained in each condition.

To reduce the effect of physiological artifacts such as breathing and cardiac activity on the INS results, we then performed a phase-scrambling permutation test^38^. Raw NIRS time series of the Yellow-Blue pairs were randomly phase scrambled, and relations between the two scrambled time series were analyzed using the same linear regression analysis. Then differences between the Beta values of the raw and the scrambled NIRS time series were assessed by *t*-test with Bootstrap (1000 times) for each ROI channel.

The ROI channels displayed significantly higher INS values for the ordered vs. scrambled time series, indicating the cortical areas showing game-related synchronization. In the competition condition, the participant pairs showed significant INS in only the right hemisphere (IPL: Ch32, *p* = 0.008; pSTS: Ch42, *p* = 0.001). In contrast, in the cooperation condition, the participants pairs showed increased INS values in the both left (IPL: Ch13, *p* = 0.002; IFG: Ch15, *p* = 0.001) and right hemispheres (pSTS: Ch39, *p* = 0.005; IFG: Ch45, *p* = 0.001).

**Figure 5:**
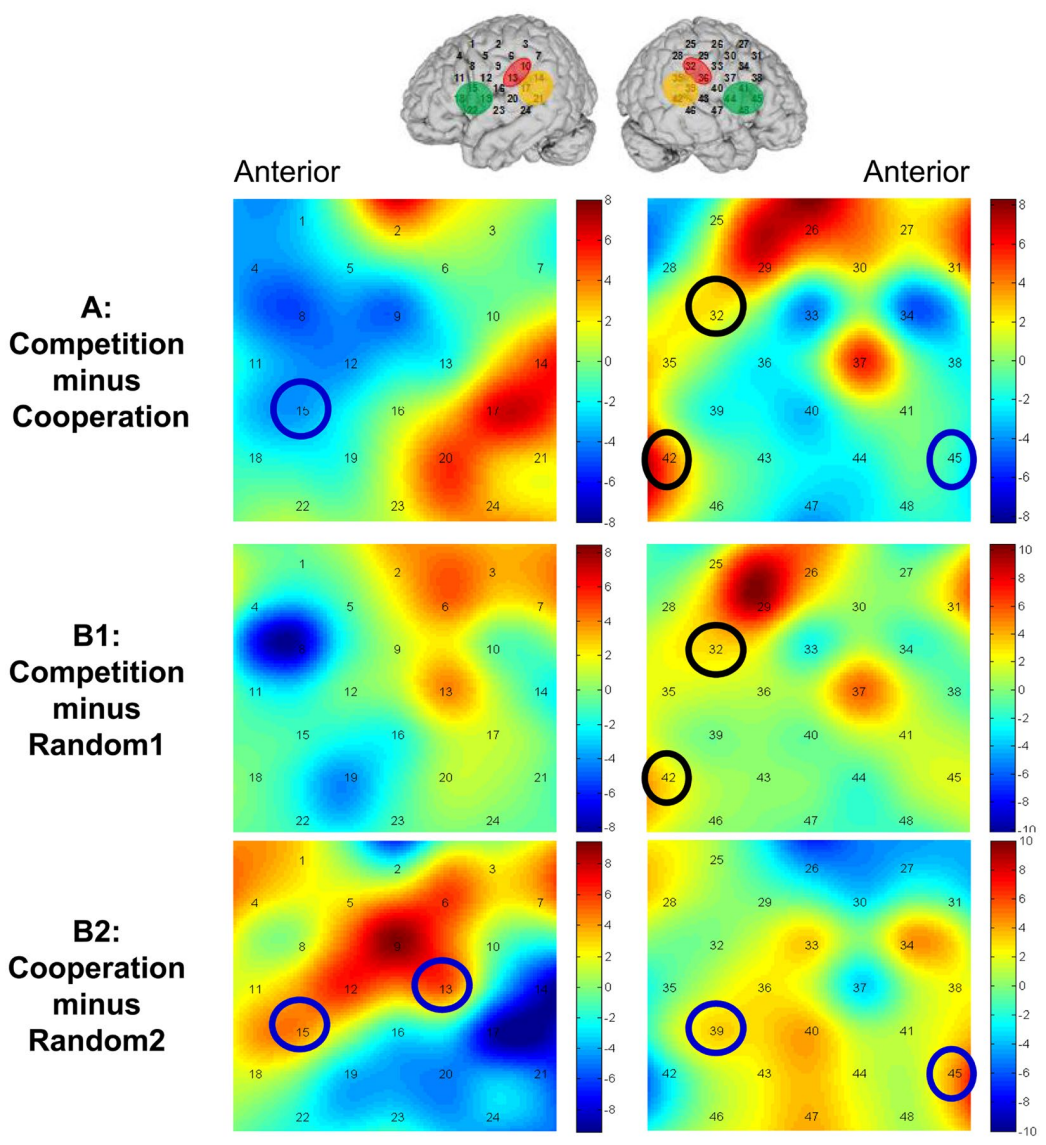
The t-test heat maps of inter-brain neural synchronization (INS) values. (**A**) Contrast result of competition condition minus cooperation condition. (**B1**) Contrast result of competition condition minus scrambled competitive baseline; and (**B2**) contrast result of cooperation condition minus scrambled cooperative baseline. The color bars show the t values. The black (blue) solid circles represent the ROI channels that showed significantly higher (*p* < 0.05) INS in the competition (cooperation) conditions, respectively.

Finally, we compared INS values of the competition and cooperation conditions using *t*-test with Bootstrap (1000 times), focusing on the above mentioned ROI channels which showed a significantly higher INS value than the scrambled time series. The result revealed that the competitive pairs showed higher INS values than the cooperative pairs in the right IPL (Ch32, *p* = 0.017) and the right pSTS (Ch42, *p* = 0.001). In contrast, the cooperative pairs showed significantly higher INS values than the competitive pairs in the bilateral IFG (Ch15, *p* = 0.001; Ch45, *p* = 0.017). Figure 5 illustrates the *t*-test heat maps of the INS values in the competition and cooperation conditions.

Taken together, these INS results suggest that the right pSTS may be commonly involved in the cooperative and competitive interactions, due to fundamental requirements of joint attention^34^ and intention understanding^36, 37^. In addition, both the intra- and inter-brain data consistently showed higher activation and INS value in the right IPL during competition than during cooperation, suggesting that the right IPL is more critical for competition. With respect to the higher INS values in the bilateral IFG during cooperation than during competition, one plausible explanation is that cooperation involves common goals and less self-other distinction, and it is thus relatively simple to achieve mutual understanding of actions and intentions.

### Relationships between empathy and performance

To assess participant pairs’ empathy trait as a coupled unit, we calculated the mean score of their PT (Perspective-Taking) and EC (Empathic Concern) ratings. Previous studies have reported that an individual’s empathy trait could promote their cooperative performance^13, 30, 31^. In the present study, however, using a linear regression analysis with PT or EC score as a regressor, we did not obtain any direct relationship between participants’ empathy and their performance for either the error rate (EC-cooperation: *p* = 0.095; EC-competition: *p* = 0.153; PT-cooperation: *p* = 0.715; PT-competition: *p* = 0.419) or the manipulation latency (EC-cooperation: *p* = 0.472; EC-competition: *p* = 0.185; PT-cooperation: *p* = 0.916; PT-competition: *p* = 0.469).

There are two possible interpretations. One is that empathy may be not necessary in the present turn-taking disk game. The alternative explanation is that, as Keysers and Gazzola have proposed, an individual’s empathy ability does not always equal to the empathic response to others^39^. For instance, an individual with a high-level of empathy ability may not show empathy to his/her opponent. A previous hyperscanning study has demonstrated significant relationship between participants’ INS value and their cooperative performance^9^. Thus, if participants showed empathic response to their partners to understand each other’s actions and intentions, they may show increased INS value, which in turn may affect their performance.

To examine the relationship between participants’ empathy and performance considering INS as mediation, we separately applied linear regression analysis between them. Specifically, we first examined whether or not the INS values in the ROI channels have significant relationship with participants’ empathy score and their behavioral performance, respectively. Then, taking both the empathy score and the INS value as two regressors (backward method), we assessed the relationship between the participants’ empathy score and their performance.

Tables 2 and 3 illustrate significant regression results in the competition and cooperation conditions. In the competition condition, participants’ EC and PT scores both significantly correlated with their INS values in the bilateral IFG, which showed significant relationships with their manipulation latency. Importantly, when we used both the empathy score and INS value as regressors, the EC score indeed contributed to their performance, but the PT score did not. In the cooperation condition, although participant pairs’ INS value in the left IFG had significant relationships with their manipulation latency and error rate (Ch15: R = 0.478, Beta = −23.897, *p* = 0.024), the EC score was not significantly correlated with manipulation latency even with the INS value as the other regressor, and there was no relationship between the participants’ empathy score and their INS in the left IFG.

**Table 2:** Linear regression results in the competition condition.

ROI	Ch	Empathy — INS						INS — Performance			Empathy + INS — Performance					
		EC			PT			Latency			EC			PT		
		R	Beta	*p*	R	Beta	*p*	R	Beta	*p*	Beta	*t*	*p*	Beta	*t*	*p*
L IFG	15	0.428	0.019	0.047	0.578	0.025	0.005	—	—	—	−13.312	−2.165	0.043	—	—	—
	18	—	—	—	0.494	0.012	0.019	—	—	—	—	—	—	—	—	—
	19	—	—	—	0.519	0.072	0.013	0.436	84.309	0.042	−15.166	−2.938	0.008	—	—	—
	22	0.577	0.046	0.005	0.570	0.043	0.006	—	—	—	−15.784	−2.315	0.032	—	—	—
L IPL	10	—	—	—	—	—	—	—	—	—	—	—	—	—	—	—
	13	—	—	—	—	—	—	—	—	—	—	—	—	—	—	—
L pSTS	14	0.479	0.155	0.024	—	—	—	—	—	—	—	—	—	—	—	—
	17	0.455	0.128	0.033	—	—	—	—	—	—	—	—	—	—	—	—
	21	0.477	0.402	0.025	—	—	—	—	—	—	—	—	—	—	—	—
R IFG	41	—	—	—	—	—	—	—	—	—	—	—	—	—	—	—
	44	—	—	—	—	—	—	—	—	—	—	—	—	—	—	—
	45	—	—	—	—	—	—	0.428	−668.45	0.047	−9.387	−1.748	0.097	—	—	—
	48	0.511	0.044	0.015	0.490	0.040	0.020	—	—	—	—	—	—	—	—	—
R IPL	32	—	—	—	—	—	—	—	—	—	—	—	—	—	—	—
	36	—	—	—	—	—	—	0.463	72.809	0.030	−15.317	−3.050	0.007	9.314	1.997	0.061
R pSTS	35	—	—	—	—	—	—	0.444	22.813	0.038	−13.729	−2.659	0.016	—	—	—
	39	—	—	—	—	—	—	0.443	33.301	0.039	−13.997	−2.710	0.014	—	—	—
	42	—	—	—	—	—	—	—	—	—	−10.503	−1.833	0.083	—	—	—

Note: INS, inter-brain neural synchronization. EC, empathic concern; PT, perspective taking. L, left; R, Right. IFG, inferior frontal gyrus; IPL, inferior parietal lobule; pSTS, posterior superior temporal sulcus.

**Table 3:** Linear regression results in the cooperation condition.

ROI	Ch	Empathy — INS						INS — Performance			Empathy + INS — Performance					
		EC			PT			Latency			EC			PT		
		R	Beta	*p*	R	Beta	*p*	R	Beta	*p*	Beta	*t*	*p*	Beta	*t*	*p*
L IFG	15	—	—	—	—	—	—	—	—	—	0.877	2.006	0.059	—	—	—
	18	—	—	—	—	—	—	—	—	—	—	—	—	—	—	—
	19	—	—	—	—	—	—	0.462	54.811	0.031	—	—	—	—	—	—
	22	—	—	—	—	—	—	—	—	—	—	—	—	—	—	—
L IPL	10	—	—	—	—	—	—	—	—	—	—	—	—	—	—	—
	13	—	—	—	—	—	—	—	—	—	—	—	—	—	—	—
L pSTS	14	0.502	0.156	0.017	—	—	—	—	—	—	—	—	—	—	—	—
	17	—	—	—	—	—	—	—	—	—	—	—	—	—	—	—
	21	0.430	0.170	0.046	—	—	—	0.409	−13.095	0.059	—	—	—	—	—	—
R IFG	41	—	—	—	—	—	—	—	—	—	—	—	—	—	—	—
	44	—	—	—	—	—	—	—	—	—	—	—	—	—	—	—
	45	—	—	—	—	—	—	—	—	—	—	—	—	—	—	—
	48	—	—	—	—	—	—	—	—	—	—	—	—	—	—	—
R IPL	32	—	—	—	—	—	—	—	—	—	—	—	—	—	—	—
	36	0.620	0.041	0.002	—	—	—	—	—	—	—	—	—	—	—	—
R pSTS	35	—	—	—	—	—	—	—	—	—	—	—	—	—	—	—
	39	—	—	—	—	—	—	—	—	—	—	—	—	—	—	—
	42	—	—	—	—	—	—	—	—	—	—	—	—	—	—	—

Note: INS, inter-brain neural synchronization. EC, empathic concern; PT, perspective taking. L, left; R, Right. IFG, inferior frontal gyrus; IPL, inferior parietal lobule; pSTS, posterior superior temporal sulcus.

Therefore, these regression results suggest two main findings. First, the mediation role of INS in relationships between empathy and performance was demonstrated in the competition condition, but not in the cooperation condition. Second, participant pairs’ empathy trait of EC, rather than the PT trait, played a major role in the present competition condition. Concerning the result of less involvement of empathy in the cooperation condition, there are two closely related explanations. One is that the cooperative game used in the present study was relatively simple for participants, and hence they could work together to copy the target pattern without empathy. The alternative possibility is that the cooperation in the present turn-taking game is a kind of “passive” cooperation, since the blue-players needed to follow the yellow-players’ move. And the passive cooperation may diminish the role of empathy.

## General discussion

The present study aimed to examine neural substrates, including intra- and inter-brain processing, underlying turn-based cooperation and competition in a real two-person situation. To achieve this goal, we simultaneously measured pairs of participants’ fronto-tempo-parietal regions using a NIRS hyperscanning technique, when they took different roles (Yellow: first-player vs. Blue: second-player) and played a computerized disk game.

Previous literature on social neuroscience has mainly focused on understanding effects of socially relevant stimuli, such as pictures or video clips containing social interactions, on the brain of a single person^5^, which cannot unravel the dynamic features of interpersonal interactions^17^. Indeed, Keysers and McKay^29^ have argued that human brain is not “an isolated stimulus-processing machine” but “a device that resonates with the brain of other individuals”. Although recent hyperscanning studies have started to examine social cognition and performance in real contexts of social interactions, most of them have mainly used task demands of body-movement synchrony, so little is known about how people taking opposing or complementary roles interact with each other in a turn-taking style^3^. Thus, the present study was designed to contribute to the hyperscanning literature by revealing neural bases of turn-based interaction. In addition, we first assessed relationships between empathy, INS, and performance, providing evidence of how empathy may improve interactive performance.

Accordingly, the main findings of the present study are two-fold. Focusing on neural bases of turn-based interaction, the manipulation latency in the present turn-taking game reflects the participant’s cognitive processing of a disk-move strategy as a so-called game plan. As expected, the latency in competition was longer than that in cooperation, indicating higher cognitive load for formulating a strategy for the game. Consistent with the behavioral data, both the intra- and inter-brain data revealed higher activation and INS value in the right IPL during competition than during cooperation. The right IPL is closely associated with cognitive functions such as perspective-taking^26^ and the self-other distinction^40, 41^. Thus, one possible explanation is that participants in the competition condition held different goals, and had relatively large uncertainty concerning their actions, leading to an increase in the computational load in order to interpret and predict each other’s disk move. In addition to the critical role of the IPL in competition, the present inter-brain analyses revealed significant INS in the right pSTS regardless of the cooperation and competition conditions, suggesting that the right pSTS may be commonly involved in both cooperation and competition, due to task demands of joint attention^34^ and intention understanding^20, 42^.

It is also noteworthy that, in our previous study^1^, participant pairs showed significant INS during competition, but not during cooperation due to the Builder-Partner role assignment, which supposedly decreased Partner’s motivation. To avoid the demotivation, we modified the instructions and the game design in the present study, and did reveal INS during the cooperation conditions in participant pairs’ right pSTS and the bilateral IFG. More importantly, the present study confirmed significant INS again in the right pSTS and the right IPL during the competition condition, suggesting the robustness of INS in turn-based competition.

A core question in social psychology and social neuroscience is how to improve interactive performance. As shown in Tables 2 and 3, the present study has revealed potential relationships between INS in the fronto-tempo-parietal regions and the competitive performance. Empathy is also a critical factor modulating social interaction^13, 30, 31^. However, inconsistent with these previous studies, we did not find any direct relationships between participants’ empathy scores and their performance. Instead, the INS value across participant pairs in their bilateral IFG was significantly correlated with empathy scores and performance. Importantly, when we took both the empathy score and INS value in the bilateral IFG as regressors, empathy showed significant relationships with the competitive performance. Keysers *et al*.^39^ and other reserachers^43^ have argued that empathic response is affected by higher cognitive functions, such as inhibitory control, attention, and motivation. Thus, self-reported empathy trait may have little direct relationships with one’s performance. One possibility is that only when individuals show a genuine empathic response to the other person, which induces an increased INS value, can empathy modulate their performance, especially in the present competition condition.

There are two main limitations in the present study. First, although we have modified the game design in the new game, the manipulation of first- and second-player still affect participants’ motivation and strategy which may desynchronize their inter-brain coupling, even though the first- and second-move is a common feature of turn-based interaction. Thus our findings from the turn-taking game task may not be generalized to other types of cooperation and competition. Second, the present study used a computerized game on a monitor. Thus, the neural correlates underlying turn-based interaction with explicit body movements such as in a real chess game should be examined in future studies.

## Conclusion

The present NIRS hyperscanning study demonstrated two main findings. The first finding relates to neural correlates of turn-based interaction. Specifically, both the cooperation and competition conditions demonstrate increased INS value in the right pSTS due to task demands of joint attention and intention understanding. As competition requires more mentalizing resources to distinguish actions of the self and the opponent, this produces a higher INS value in the right IPL than during cooperation. Second, there may be no direct relationship between participant dyads’ empathy and performance, which is mediated by the INS between interacting persons.

## Materials and Methods

### Participants

Forty-four (22 pairs) male students (age: 19.0 ± 1.4 years) from Nagoya University took part in the present study for course credit. All participants were right-handed (handedness score: 89.2 ± 11.4) as assessed by the Edinburgh Handedness Inventory^44^, and had normal or corrected-to-normal vision. Since friendship as an important factor may influence the result of inter-brain correlation, we assessed their friendship using a self-report questionnaire after the experiment (friendship: 0.1 ± 0.1 years). They were informed about the purpose and safety of the experiment, and written informed consent was obtained from all participants, who were native Japanese and naive to the purpose of the study. All methods were carried out in accordance with the principles and guidelines of the Declaration of Helsinki, and all experimental protocols were approved by Institutional Review Boards of Nagoya University. In addition, participants were also instructed that their photos would be only used in academic ways and all their personal information would be restrictively protected. Finally, the informed consent on the usage of their photos was obtained.

### Materials and design

A computerized two-person game was used in the present study to present participants with cooperative and competitive contexts. Pairs of participants sitting side-by-side played the game in a turn-taking style under two experimental conditions and one control condition. In the cooperation condition, participant pairs were instructed to control different colored disks (yellow and blue) to collaboratively copy a target pattern (i.e., occupying all of the target positions) presented on a monitor, while in the competition condition, they were asked to occupy as many target positions as possible to defeat their partner. To assess the homogeneity of the brain activity in the two groups of participants, they also played the game independently with the other participant watching quietly sitting beside as a control condition.

The target pattern, varying across different games, was composed of five yellow disks within a three-by-three matrix. In a game, participant pairs were given a total of eight disks, i.e., four disks per participant, to achieve their cooperative or competitive goals. The participant who controlled yellow disks (yellow player) always took the initial turn as the first-player, and the other participant controlling blue disks (blue player) followed as the second-player. Two yellow dots were pasted on the keys “1” and “3”, and the yellow-players were asked to press the two dots using their index and middle fingers to control the horizontal moves of the yellow disks. The “1” key moved the disk to the left and the “3” key moved it to the right. The blue-players were likewise instructed to press two blue dots that were pasted on the keys “4” and “6” to control the blue disks. Each participant was given 2 s for their disk control in one turn, within which (s)he could move the disk left and right as many times as possible. A game began with the appearance of the yellow disk at the top left of the display. When holding down the key, the disk moved at a constant speed from column to column until the key was released or 2 s had elapsed. Then the disk dropped quickly to the lowest available space in that column. Simultaneously with this movement, the next disk (blue one) appeared at the top left of the display signaling the start of the second player’s turn.

### Experimental procedures

Prior to the experiment, participant pairs received training on three types of games (i.e., cooperation, competition, and independent games) lasting approximately 3–5 min, until they sufficiently understood the game and played without errors. And then, they played 48 games (16 games in each condition) in two runs (about 20 min) with measurement of their brain activations using NIRS. Each experimental run consisted of 12 game-sets, in which two same-type games were presented continuously. In addition, two resting-sets were assigned respectively in the middle and at the end of the game-sets. The order of the game-sets was pseudo-randomized to ensure that no two adjacent game-sets were the same type.

Before the first game-set, a 10-s black screen was displayed. And all game-sets were preceded by a 3-s instruction of the game type (e.g., the message “The following session is Cooperation!” was displayed on monitor) and a 1-s black screen. After each game-set, another 3-s black screen was presented as an inter-block-interval. The two continuous games within one set were separated by a 1-s black screen (for details of the temporal structure, see supplementary material Figure a). Two seconds were given to participants to move each disk, resulting in a total of 16 sec for one game.

The whole experimental procedure was recorded by two Digital Videos. And the disk manipulations by players were automatically recorded by the game program. After the experiment, participants’ friendship and empathy trait were assessed by a self-report questionnaire and a four-scale Interpersonal Reactivity Index questionnaire, respectively.

### Apparatus

A 96-channel NIRS system (LABNIRS; Shimadzu Co., Japan) was employed to measure concentrations changes of oxygenated hemoglobin (HbO), deoxygenated hemoglobin, and total hemoglobin in participant pairs’ brains simultaneously. A channel was defined as the middle part of one emitter optode and one detector optode located 3 cm apart. For one participant, thirty-two optodes (16 emitters and 16 detectors) were bilaterally placed on the frontoparietal regions in a lattice pattern, forming 24 channels for each hemisphere (9 × 9 cm^2^). The middle channels in the lowest line were located respectively at the T3 and T4 positions according to the international 10–20 system. The sampling rate for measurement was 37 Hz.

Based on the 3-dimensional probabilistic anatomical craniocerebral correlation, the T3 and T4 were projected onto the left and right middle temporal gyrus^45^. Thus, the NIRS system approximately covers bilateral fronto-tempo-parietal regions in close proximity to scalp tissues. Specifically, positions of all the NIRS channels were measured by a 3D electromagnetic tracking device (FASTRAK; Polhemus, USA) after the experiment with three different subjects, and then were registered on the Montreal neurological Institute (MNI) brain space using a virtual registration method^46^. Finally, the estimated mean locations of the NIRS channels were obtained using anatomical information based on Brodmann areas. As regions of interest, we mainly focused on the channels approximately covering the IFG (left: Ch15, Ch18, Ch19, Ch22; right: Ch41, Ch44, Ch45, Ch48), the pSTS (left: Ch14, Ch17, Ch21; right: Ch 35, Ch39, Ch42) and the IPL bilaterally (left: Ch10, Ch13; right: Ch32, Ch36).

### Data analysis

For the NIRS data, only the HbO was analyzed since oxygenated hemoglobin is the most sensitive parameter of regional cerebral blood flow and provides a robust correlation with the BOLD signal of fMRI^47^. The individual NIRS data were analyzed independently for each ROI. Sampling rate of the NIRS raw data was reduced to 10 Hz, and motion artifacts by head movements were eliminated. Then a baseline correction was applied using mean HbO during the 2-s black-screen period before each game-set to remove any longitudinal signal drift. NIRS data are originally relative values, rather than absolute values like fMRI, and hence cannot be averaged directly across the channels and participants. To address this issue, the NIRS data of each game-set after baseline correction were converted to *z*-scores by the preceding 2-s baseline^1, 2^.

Finally, the NIRS data were analyzed in two different ways, depending on whether intra-brain activation or inter-brain neural synchronization was focused on. Concerning analysis of intra-brain activation, group-averaged data were calculated across 16 games of each type of condition. And then a two-way ANOVA [Role (yellow vs. blue) × Condition (cooperation vs. competition)] was conducted on mean HbO in each ROI, independently.

To assess the issue of INS across participant pairs, the *z*-scores in one game were averaged every 0.5-s forming 32 points. And then we analyzed two NIRS time series of Yellow-Blue pairs over consecutive 16 s game rounds for each pair in each condition using linear regression analysis with game timing as a regressor. To reduce the physiological effect, we further performed a phase-scrambling permutation test^38^, and mainly focused on the channels which showed a higher INS value than the scrambled time series. Furthermore, we also analyzed relations among participants’ empathy, INS, and performance using a backward linear regression method. All the statistical analyses were carried out using Statistical Package for the Social Sciences 19 (SPSS). The significance level was set at *p* < 0.05.

## Electronic supplementary material

Supplementary Video 1

Supplementary Video 1-caption
