# Supplementary material for: Inter-brain network underlying turn-based cooperation and competition: A hyperscanning study using near-infrared spectroscopy
Source: Sci Rep. 2017 Aug 17;7:8684. doi: 10.1038/s41598-017-09226-w (PMC5561070; doi:10.1038/s41598-017-09226-w)
Supplement: Supplementary file 2 — Supplementary Video 1-caption [file 41598_2017_9226_MOESM2_ESM.doc]

**Inter-brain network underlying turn-based cooperation and competition: A hyperscanning study using near-infrared spectroscopy**

**Tao Liu^1,3^, Godai Saito**^4^**, Chenhui Lin^2^, and Hirofumi Saito^2,*^**

^1^ School of Management, Zhejiang University, Hangzhou 310058, China

^2^ Department of Cognitive Informatics, Graduate School of Informatics, Nagoya University, Furo-cho, Chikusa-ku, Nagoya 464-8601, Japan

^3^ Department of Psychology, Sun Yat-Sen University, Guangzhou 510275, China

^4^ Department of Psychology, Graduate School of Arts and Letters, Tohoku University, 27-1 Kawauchi, Aoba-ku, Sendai 980-8576, Japan.

^*^ Corresponding author.

E-mail address: saito@is.nagoya-u.ac.jp

Tel: + 81 052 789 5550; fax: + 81 052 789 5452.

Supplementary Video 1. Example of video clip of a cooperation game in the present study.
